# Clinical and Epidemiological Features of Calicivirus Infections in Cattle

**DOI:** 10.3390/ani16050829

**Published:** 2026-03-06

**Authors:** Krisztián Bányai, Valantine Ngum Ndze, Ágnes Bogdán, Attila Kiss, Tamás Tóth, Zsófia Lanszki, Gianvito Lanave, Francesco Pellegrini, Barbara Di Martino, Vito Martella

**Affiliations:** 1 Department of Medical Biology, Medical School, University of Pécs, 7624 Pécs, Hungary; agnesbogdan@gmail.com; 2 Department of Pharmacology and Toxicology, University of Veterinary Medicine, 1078 Budapest, Hungary; vito.martella@uniba.it; 3 National Laboratory of Infectious Animal Diseases, Antimicrobial Resistance, Veterinary Public Health and Food Chain Safety, HUN-REN Veterinary Medical Research Institute, 1143 Budapest, Hungary; 4 Faculty of Health Sciences, University of Buea, Buea P.O. Box 63, Cameroon; valentinengum@yahoo.com; 5 Agricultural and Food Research Center, Széchenyi István University, 9026 Győr, Hungary; kiss.attila.peter@sze.hu (A.K.); toth.tamas@sze.hu (T.T.); 6 National Laboratory of Virology, Szentágothai Research Centre, University of Pécs, 7624 Pécs, Hungary; lanszkizsofi@gmail.com; 7 Institute of Biology, Faculty of Sciences, University of Pécs, 7624 Pécs, Hungary; 8 Department of Veterinary Medicine, University of Bari Aldo Moro, 70010 Valenzano, Italy; gianvito.lanave@uniba.it (G.L.); francesco.pellegrini@uniba.it (F.P.); 9 Department of Veterinary Medicine, Università degli Studi di Teramo, Località Piano D’Accio, 64100 Teramo, Italy; bdimartino@unite.it

**Keywords:** norovirus, nebovirus, vesivirus, zoonosis

## Abstract

The family *Caliciviridae* encompasses a diverse group of non-enveloped, positive-sense RNA viruses that are significant pathogens in veterinary medicine. This narrative review summarizes the current state of knowledge regarding the clinical, molecular, and epidemiological features of the three calicivirus genera identified in bovine hosts: *Norovirus*, *Nebovirus*, and *Vesivirus*. Bovine noroviruses and neboviruses are neglected enteric pathogens, frequently detected in association with neonatal calf diarrhea and often present in co-infections with other enteric agents. Clinical presentations for these enteric viruses range from severe, watery diarrhea to asymptomatic shedding, with distinct pathogenic profiles observed between norovirus genotypes GIII.1 and GIII.2. In contrast, the genus *Vesivirus* exhibits a broad host range, and bovine vesivirus strains are phylogenetically linked to vesiviruses identified in pigs and marine animals. Bovine vesivirus infections are associated with a broader spectrum of clinical manifestations, including respiratory disease, vesicular lesions, and abortion. Serological and virological surveys indicate that exposure to these viruses is ubiquitous in cattle populations globally. While direct evidence of human infection by bovine noroviruses and neboviruses remains limited, vesiviruses possess a confirmed capacity for cross-species transmission to humans. Significant knowledge gaps remain, particularly regarding in vitro culture systems, necessitating further research to facilitate vaccine development and clarify transmission dynamics.

## Simple Summary

Caliciviruses are a family of small viruses that infect many different animals, including cattle. Three specific types (genera) of caliciviruses infect cows: *Norovirus*, *Nebovirus*, and *Vesivirus*. Both bovine noroviruses and neboviruses are important causes of diarrhea in young calves, often contributing to a condition known as neonatal calf diarrhea. Bovine vesiviruses are related to viruses found in marine mammals and pigs and can cause respiratory problems, blisters (vesicles), and abortions in cattle. Caliciviruses are found in cattle herds globally. Importantly, some bovine caliciviruses have zoonotic implications. While vesiviruses are known to infect humans, the risks posed by bovine noroviruses and neboviruses are still being investigated. Further investigation is needed to define the mechanisms of viral spread between homologous and heterologous hosts.

## 1. Introduction

The family *Caliciviridae* includes a large group of small, non-enveloped viruses harboring a positive-sense, single-stranded RNA genome [1]. The viral particles are typically 27–40 nm in diameter and are characterized by icosahedral symmetry displaying cup-shaped depressions (calices) on their surface, a feature from which the family name is derived. The genomic RNA, with 6.4 to 8.5 kb in length, encodes a major capsid protein (VP1), a minor capsid protein (VP2), and non-structural proteins playing key roles in viral replication. The number of open reading frames (ORFs) along the genome varies among genera and ranges from 2 to 4. The family currently includes 11 approved genera: *Vesivirus*, *Norovirus*, *Sapovirus*, *Lagovirus*, *Nebovirus*, *Recovirus*, *Valovirus*, *Bavovirus*, *Nacovirus*, *Minovirus*, and *Salovirus* [1]. This recent expansion of calicivirus taxonomy reflects the widespread use of genome sequencing technologies, which have allowed for a more complex understanding of viral diversity beyond earlier morphology-based identification. Caliciviruses infect all major groups of vertebrates, including fish, amphibia, reptiles, birds and mammals [1].

Domestic ruminants are known to harbor representative members of three genera, such as *Norovirus*, *Nebovirus* and *Vesivirus* (Table 1). Members of the genera *Norovirus* (e.g., Jena virus) and *Nebovirus* (e.g., Nebraska virus) are neglected bovine pathogens. Bovine norovirus and bovine nebovirus infections are commonly identified in neonatal calf diarrhea (NCD), a multifactorial syndrome, and are thought to function as opportunistic pathogens under certain conditions. The genus *Vesivirus* is characterized by an exceptionally broad host range, a feature that distinguishes it from other calicivirus genera, which typically exhibit more restricted host specificity. In cattle, vesivirus infection is rarely identified but the observed clinical manifestations can be diverse [2,3,4,5,6,7,8,9,10,11].

This narrative review summarizes the main features of calicivirus infections in cattle, focusing on the clinical and epidemiological features. Due to the frequent contact between these livestock animals and humans, we present an overview of the zoonotic potential of bovine caliciviruses.

## 2. Bovine Norovirus Infections

The prototype norovirus, designated Norwalk virus, was described by Kapikian and colleagues in 1972 in a gastroenteritis outbreak at an elementary school in Norwalk, Ohio, USA, in 1968 [12]. Shortly after, veterinary researchers reported the identification of morphologically similar virus particles in the feces of diarrheic calves. In the United Kingdom, Woode and Bridger while in Germany, Gunther and Otto, described bovine enteric caliciviruses, Newbury agent 2 (Bo/Newbury2/76/UK) and Jena virus (Bo/Jena/80/DE), respectively [5,7]. These viruses were isolated from diarrheic calves and later molecular characterization classified both viral agents as bovine representatives of noroviruses [6,13].

In conjunction with electron microscopy, experimental infections in susceptible gnotobiotic (Gn) or colostrum-deprived calves were crucial for establishing the etiological link between these viral agents and the observed enteric disease [14]. The near-simultaneous discovery and morphological description of similar viruses in humans and cattle implicitly suggested the existence of a family of viruses with similar structures capable of infecting different mammalian hosts, laying conceptual groundwork for later molecular studies.

### 2.1. Molecular Biology and Classification

**Figure 1:**
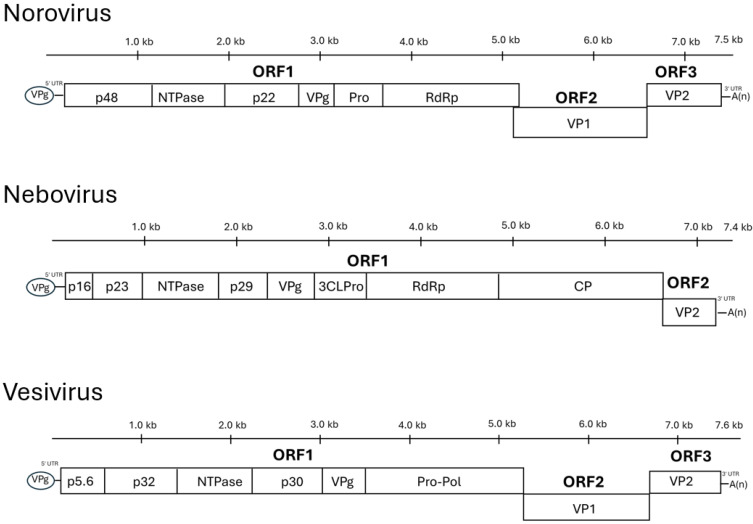
Calicivirus genomes (*Norovirus*, *Nebovirus*, and *Vesivirus*) consist of positive-sense ssRNA featuring a 5′ VPg and a 3′ poly(A) tail. In all genera, ORF1 encodes a polyprotein processed into non-structural units: N-terminal proteins (p48, p16/p23, or p5.6/p32), NTPase, intermediate proteins (p22, p29, or p30), and VPg. While noroviruses and neboviruses utilize distinct Pro/3CLPro and RdRp enzymes, vesiviruses express a fused Pro-Pol precursor. The primary architectural distinction lies in capsid encoding: noroviruses and vesiviruses utilize three ORFs, with the major (VP1) and minor (VP2) capsids in ORF2 and ORF3. Conversely, neboviruses feature only two ORFs, uniquely translating its major capsid (CP) within the ORF1 polyprotein, leaving ORF2 to encode VP2.

The norovirus genome, without the 3′ poly(A) tail, is approximately 7.3 to 7.5 kb in length and contains three open reading frames (ORFs) [6,15]. ORF1 is located at the 5′ end of the genome. It encodes a large polyprotein (approximately 180 kDa in Jena virus) that undergoes autocatalytic proteolytic cleavage by the viral 3C-like protease. This processing yields six nonstructural proteins that are involved in viral replication, including a N-terminal protein (p48), a NTPase, a 3A-like protein (p22), a VPg, a 3C-like protease, and an RNA-dependent RNA polymerase (RdRp). ORF2 encodes the major structural capsid protein (55–60 KDa), VP1, which expresses the main antigenic determinant. ORF3 encodes a small, basic minor structural protein (20–25 KDa), VP2, which is believed to play a role in virion stability and assembly. A fourth ORF (ORF4) has been described for the homologous murine norovirus (not for bovine noroviruses); this gene encodes a virulence factor (VF1) that modulates the host innate immune response [16,17] (Figure 1).

Noroviruses belong to family *Caliciviridae*, genus *Norovirus*, and species *Norovirus norwalkense* [1]. *Norovirus norwalkense* (the type species, formerly known as *Norwalk virus*) is the single species within the *Norovirus* genus. Noroviruses exhibit significant genetic diversity and are classified into at least ten genogroups, designated GI through GX, based on phylogenetic analysis of the complete amino acid sequence of the major capsid protein, VP1 [18]. These genogroups can be further subdivided into numerous genotypes.

Bovine noroviruses (BNoVs) are primarily classified within Genogroup III (GIII) [19]. This genogroup also includes noroviruses found in sheep (ovine noroviruses) [20]. Within GIII, several distinct genotypes relevant to cattle have been identified. For genotype GIII.1, the prototype strain is Jena virus (Bo/NLV/Jena/80/DE), whereas for genotype GIII.2, the prototype strain is Newbury2 virus (Bo/NLV/Newbury2/76/UK) [21]. This genotype is frequently reported as predominant in many cattle populations. Other genotypes within GIII have been detected in closely related ungulates; for example, genotype GIII.3 seems to be associated with ovine strains, whereas genotype GIII.4 has been identified in yaks and cattle in China, based on partial genome sequences [19,22,23,24].

The current virus nomenclature is affected by genomic recombination, which is a major evolutionary driving force for noroviruses and contributes to the observed viral genetic diversity [25]. Recombination events are very often reported in epidemiologic studies, particularly at the junction between ORF1 (encoding the polymerase) and ORF2 (encoding the capsid protein) [26]. This process can generate novel strains with a polymerase region and a capsid region derived from different parental viruses. For example, in this dual nomenclature system, the term GIII.P1/GIII.2 indicates a GIII polymerase type 1 and a GIII capsid type 2. In fact, several such recombinant BNoV strains have been identified in field studies [27,28,29]. This dual-typing system enables precise tracking of the emergence and spread of novel strains with potentially altered biological features.

### 2.2. Clinical Features and Pathology

BNoVs are primarily recognized for their association with enteric disease in cattle, particularly in young calves. In field settings, BNoV is frequently detected in calves with diarrhea, often as part of the neonatal calf diarrhea (NCD) complex [19,30,31]. BNoV-associated diarrhea is typically watery and non-hemorrhagic and other clinical signs may include anorexia, lethargy, and malabsorption. Body temperature remains often normal. BNoV infections can occur as the sole identified pathogen in an affected calf, but they are commonly found in co-infections with other enteric pathogens, including rotavirus, coronavirus, bacteria, and protozoa [30,31].

Experimental infections have used well-characterized BNoV strains and newborn Gn or colostrum-deprived commercial calves [32,33]. These experiments revealed genotype-specific differences in clinical presentation and pathology (Table 2).

For example, oral inoculation of calves with GIII.1 BNoV strains (e.g., Jena virus) reproducibly induces severe, watery enteric disease. Diarrhea typically appears rapidly (14–16 h post-inoculation, hpi) and lasts about 2–3 days [32]. GIII.1 BNoV infection causes significant small intestinal lesions, characterized by severe villus atrophy, villus fusion, and loss of absorptive epithelial cells, most prominently in the jejunum and ileum. Crypt hyperplasia is also common. Viral capsid antigen is found in affected villus epithelial cells. The extensive cell loss might paradoxically shorten the infection duration by rapidly depleting susceptible target cells. Viral shedding in GIII.1 experimental infections is detectable early (12 hpi) and typically ceases as early as 23 hpi or can extend up to 4 days post-infection (dpi). The clinical signs and intestinal pathology induced by GIII.1 BNoV in calves are considered comparable to those observed in human norovirus infections, making this bovine model valuable for studying general norovirus pathogenesis.

In contrast, experimental infections with GIII.2 BNoV strains often result in a different clinical and pathological picture. Conventionally reared calves inoculated with GIII.2 (Newbury virus) show little to no diarrhea. In Gn calf models, GIII.2 strains (e.g., Newbury, CV186-OH/00/US) typically induce milder clinical signs compared to GIII.1, including mild diarrhea, transient anorexia, and xylose malabsorption [33]. Diarrhea in these Gn calves can persist for extended periods, up to 20–26 dpi. GIII.2 infections, unlike GIII.1, do not necessarily induce significant histological changes like severe villus atrophy or extensive intestinal cell death. Pathogenesis is generally characterized by mild enteropathogenicity. A prominent feature of GIII.2 BNoV infection is prolonged fecal viral RNA shedding, often detectable for at least 20–30 dpi, with peak titers early in infection. This prolonged shedding, even without severe clinical signs, is considered a significant factor in the persistence and endemic circulation of GIII.2 strains in cattle populations [33].

Collectively, while results from Gn calf studies are relevant for understanding primary viral effects and mechanisms of pathogenesis, they should be extrapolated with caution to field situations where host and environmental factors are considerably more complex. Both Gn and conventional calf models provide complementary and valuable information. For instance, GIII.2 (Newbury virus) was reported to cause little or no diarrhea in conventionally kept calves, whereas the GIII.2 CV186-OH strain induced mild, persistent diarrhea in Gn calves [33].

### 2.3. Epidemiological Features

Epidemiological investigations of bovine norovirus (BNoV) rely on two main approaches: the direct detection of the virus (or its components, typically RNA) in samples and the detection of host antibodies against the virus in serum. Together, these methods provide insights into the current circulation, geographical distribution, and historical exposure of cattle populations to BNoV.

#### 2.3.1. Prevalence, Genotypes

BNoVs have a worldwide distribution; regions and countries reporting BNoV circulation include North and South America, Europe, Asia, and Africa (Table 3) [34,35,36,37,38,39,40,41,42,43,44,45,46,47,48,49,50,51,52]. The primary diagnostic tools for virological surveillance are molecular methods, predominantly endpoint or quantitative RT-PCR. These assays are typically designed to target conserved regions within the BNoV genome, such as the RNA-dependent RNA polymerase (RdRp) gene in ORF1, or regions within the capsid gene (ORF2), or a combination of primers spanning both ORFs [19].

Reported virological prevalence rates of BNoVs in cattle vary significantly, from <1% to as high as 80% in different studies [34,51]. This wide range is due to genuine epidemiological differences between regions, but also variations in study design (e.g., targeting diarrheic versus clinically normal calves, age groups, breeds), and the sensitivity and specificity of the diagnostic assays employed. For example, a study in South Korea found BNoV in 9.3% of diarrheic fecal samples using nested PCR, but in only 2.8% of the samples using a single-step RT-PCR [45]. Likewise, some RT-PCR protocols may exhibit specificity failures for certain genotypes, potentially underestimating their prevalence if not broadly reactive. These methodological variations make direct comparisons challenging and underscore the need for standardized diagnostic approaches.

Genotype GIII.2 (Newbury2-like) is frequently reported as the predominant BNoV genotype in many countries, likely due to its prolonged shedding, which increases transmission and environmental persistence [24]. However, GIII.1 (Jena-like) strains are also detected, and there is increasing recognition of recombinant BNoV strains (e.g., GIII.P1/GIII.2, GIII.P2/GIII.1) circulating in cattle populations [27]. In China, for instance, GIII.1 strains have been reported, and a novel putative GIII.4 genotype (GIII.P2/GIII.4) has been identified in yaks and dairy cows [22,23,50].

BNoV infections are often more frequently detected in younger compared to older calves. BNoV is commonly found in fecal samples from diarrheic calves, supporting its role as an enteric pathogen. However, the virus is also frequently detected in non-diarrheic, clinically healthy animals, indicating common subclinical infections and asymptomatic shedding [19]. This complicates the assessment of BNoV direct contribution to NCD in individual field cases, particularly when other pathogens are present. BNoV is often found in co-infections with rotavirus, nebovirus, pathogenic *Escherichia coli*, and *Cryptosporidium parvum* [53].

Serological surveys complement virological studies by providing evidence of past exposure to BNoV and an indication of the extent of viral circulation within cattle populations over time. Serological investigations conducted in various parts of the world consistently reveal very high exposure rates to BNoV in cattle. These studies primarily employ enzyme-linked immunosorbent assays (ELISAs) that utilize virus-like particles (VLPs) (typically from GIII.1 Jena virus or GIII.2 Newbury2 virus) as the target antigen [38]. Extremely high seroprevalence rates, often exceeding 90%, have been reported in many countries for one or both major GIII genotypes. Examples include Germany (68.5% for GIII.1 and 90.5% for GIII.2), Belgium (93.2% for GIII.2), UK (87.5% for GIII.1 and 76.5% for GIII.2), and the USA (94–100% for GIII.2) [54,55,56]. This strongly suggests that BNoV infection is ubiquitous and endemic in most cattle populations worldwide, implying early life exposure for the vast majority of cattle.

Serological studies have shed light on the dynamics of antibody responses in relation to age [54]. In calves, antibody levels against GIII.2 BNoVs tend to rise significantly during the first six months of life, following the decline of maternally derived antibodies, and these antibody levels are generally maintained into adulthood [38]. This pattern is indicative of widespread infection occurring early in life. The continued presence of antibodies and the potential for antibody titers to rise again in adult cattle suggest that re-infections are common throughout life [38]. This phenomenon, coupled with the genetic and antigenic diversity of BNoV, suggests that immunity following natural infection may be short-lived or strain- or genotype-specific, as observed with human norovirus infections. This poses a challenge for vaccine development, as an ideal vaccine would need to confer broad and durable protection against multiple circulating strains and genotypes.

Evidence indicates that BNoV GIII.1 (Jena) and GIII.2 (Newbury2) are antigenically distinct [57]. ELISAs using convalescent calf sera against one genotype show little to no cross-reactivity with VLPs from the heterologous genotype, though at least one cross-reactive epitope has been identified. This antigenic distinctness highlights the importance of using genotype-specific antigens (VLPs) in ELISAs for accurate determination of exposure to specific BNoV genotypes.

The marked difference in pathogenicity and shedding duration between GIII.1 (Jena-like) and GIII.2 (Newbury2-like) strains observed in experimental settings has significant implications for understanding field epidemiology and for the interpretation of diagnostic results. While GIII.1 strains appear to cause more acute and severe disease, their shedding period may be relatively short [32]. Conversely, GIII.2 strains, though often associated with milder or even subclinical disease, can be shed for prolonged periods. This extended shedding window for GIII.2 could lead to greater environmental contamination and more opportunities for transmission, potentially explaining why GIII.2 is often reported as the more prevalent genotype in many field surveillance studies [33].

#### 2.3.2. Zoonotic Potential

The zoonotic potential of BNoVs is currently considered a minor public health concern. While BNoVs are primarily classified in genogroup GIII, distinct from the main human-pathogenic genogroups (GI, GII, GIV), all noroviruses share a common ancestry, and other animal noroviruses (e.g., from pigs, dogs, cats) cluster within human-prominent genogroups like GII and GIV [58].

**Figure 2:**
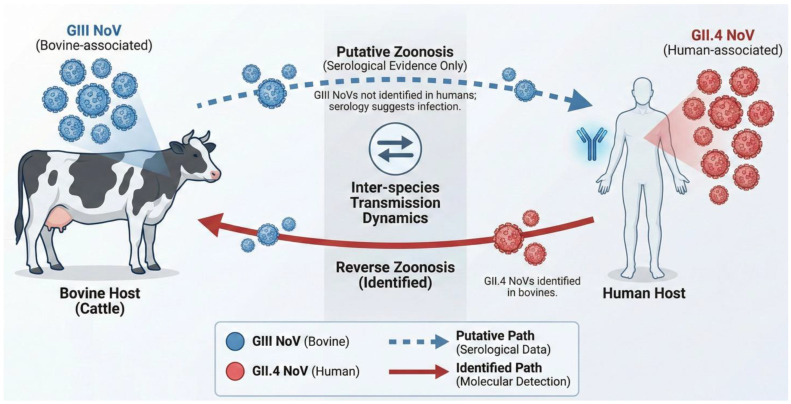
Putative transmission pathways between bovine and human hosts for GII.4 and GIII noroviruses.

Direct evidence regarding the role of cattle in the broader norovirus ecosystem includes the detection of human-pathogenic norovirus genotypes, such as the GII.4 Sydney strain, in bovine (and swine) fecal samples in Canada [58]. This suggests that cattle can be infected with and potentially shed human strains, acting as reservoirs. Experimental infections of Gn calves with human GII.4 norovirus also showed viral replication and immune response (Figure 2) [59].

The attachment of noroviruses to host cells is a multi-step process initiated by the recognition of histo-blood group antigens (HBGAs), which are complex carbohydrates found on the surface of intestinal epithelial cells [60]. While GIII BNoVs typically target α-galactose residues rare in humans, dominant human strains like GII.4 exhibit a flexible binding pocket that allows them to “dock” onto bovine intestinal tissues [60]. Research using Gn calves has shown that human GII.4 strains specifically target the villous epithelial cells of the duodenum and jejunum [59]. This attachment is primarily mediated by terminal sugars such as α1,2-linked fucose, a core component of H-type antigens that are conserved across many mammalian species. This shared carbohydrate niche between humans and cattle could facilitate the persistence of human noroviruses in animals and highlights the risk of cattle serving as passive reservoirs [60].

Serological studies provide indirect evidence of human exposure to animal noroviruses. Antibodies against GIII BNoVs have been found in human sera. For example, a study in the Netherlands found higher seroprevalence of GIII.2 antibodies in veterinarians (28%) compared to the general population (20%), suggesting occupational exposure [61]. Anti-GIII antibodies were also detected in adult blood donors in Sweden, and in an Indian birth cohort [62,63]. While antibody cross-reactivity should not be ruled out, some studies indicate that not all anti-GIII.2 human antibody responses are solely due to cross-reactivity with human GI or GII strains [61].

Because noroviruses frequently undergo genetic recombination, particularly at the ORF1/ORF2 junction, a key driver of their evolution, the co-circulation of human and animal noroviruses raises concern that co-infection could lead to the emergence of recombinant viruses with altered host tropism, virulence, or transmissibility, potentially making cattle “mixing vessels” for novel norovirus strains. Despite all this evidence, no definitive reports confirm a bovine-specific GIII norovirus as the causative agent of human gastroenteritis outbreaks. While GIII noroviruses have not typically caused human disease, the potential for zoonotic transmission through recombination or adaptation of human strains in animal reservoirs awaits formal demonstration [64].

## 3. Bovine Nebovirus Infections

The Newbury agent 1 (NA1), officially designated Bo//Newbury1/1976/UK, was identified in 1976 from diarrheic calves in the United Kingdom [8]. Initial characterization of NA1 based on electron microscopy revealed particles approximately 36 nm in diameter with cup-like surface depressions, a buoyant density of ~1.34 g/cm^3^, and the presence of a single major capsid protein with a molecular mass of about 49 kDa [65]. These features were consistent with those of the *Caliciviridae* family. In the United States (US), the Nebraska (NB) strain (e.g., Bo/Nebraska/80/US) was characterized and, along with NA1, became a prototype for the viruses that would later form the *Nebovirus* genus. The formal recognition of these distinct bovine caliciviruses culminated in the official classification of *Nebovirus* as a new genus within the *Caliciviridae* family by the ICTV in 2010 ([9], https://ictv.global/ictv/proposals/2008.123-126V.v2.Nebovirus.pdf, accessed on 5 January 2006).

### 3.1. Molecular Biology and Classification

The nebovirus genome consists of a linear, positive-sense, single-stranded RNA molecule, typically around 7.4 to 7.5 kilobases (kb) in length (Figure 1); for example, the NA1 strain genome is 7454 nucleotides (nt) and the NB strain genome is 7453 nt [9,10]. A viral protein, known as VPg (virion protein, genome-linked), is covalently attached to the 5′-terminus of the genomic RNA, and a polyadenylated (i.e., poly(A)) tail is present at the 3′-terminus. The VPg protein is essential for viral replication and is understood to play a critical role in the initiation of translation. The genomic organization of *Nebovirus* is characteristic and distinguishes it from some other calicivirus genera. Neboviruses, along with selected viruses within *Caliciviridae* possess two main open reading frames (ORFs) [9,10]. ORF1 is a large, 5′-proximal ORF encodes a polyprotein. This polyprotein is co- and post-translationally cleaved by the virus-encoded 3C-like protease (NS6pro) into several mature non-structural (NS) proteins. These NS proteins include, an N-terminal protein (NS1/2 or p30), an NTPase/helicase (NS3), a protein of unknown function (NS4 or p39), the VPg protein (NS5), the protease (NS6pro), and the RNA-dependent RNA polymerase (RdRp or NS7). Similar to some other two-ORF caliciviruses, a key feature of *Nebovirus* is that the coding sequence for VP1 is located at the 3′-end of ORF1 and is contiguous with the NS polyprotein sequence. ORF2 is the second ORF, located downstream of ORF1, and typically encodes the minor structural protein VP2. During viral replication, a subgenomic RNA is produced. This subgenomic RNA is 3′-co-terminal with the genomic RNA and serves as an efficient template for the translation of the structural proteins (VP1 and VP2), ensuring ample production of capsid components for virion assembly [9,10].

The genus name, *Nebovirus*, evolved over time. For taxonomic accuracy and clarity in scientific communication, *Nebovirus* is the current genus designation, while “*Becovirus*” is best understood as a significant historical proposal that highlighted the unique nature of these viruses before their formal classification. The more general term “Bovine enteric calicivirus (BECV)” can be seen as a descriptive term, though it was also specifically linked to NA1.

The *Nebovirus* genus exhibits considerable genetic diversity. Several distinct strains and potential genotypes or clades have been identified globally. These include NB-like strains, which are widely reported from various countries, NA1-like strains which are also found globally, representing another distinct lineage. Some novel strains, including Bo/DijonA216/06/FR strain identified in France or Kirklareli strain from Türkiye, Bo/YLA-2/17/CH from China, and BNeV/Mukti/2016/IND strain from India, are thought to represent novel, yet unclassified, nebovirus genotypes [44,66,67,68]. The ongoing discovery of such new strains and genetic variants, including recombinants, indicates that the genetic landscape of the *Nebovirus* genus is still being actively explored and is likely more diverse than initially recognized.

### 3.2. Clinical Features and Pathology

Bovine neboviruses (BNeV) are primarily linked to enteric disease in neonatal calves [9,10]. The most common signs are watery, profuse, and persistent diarrhea, often accompanied by dehydration, toxemia, and enophthalmos (sunken eyes) due to fluid loss. Affected calves may also show anorexia, lethargy, and depression. In some herds, BNeV infections have been associated with calf mortality rates of up to 30%. BNeVs are frequently found in co-infections with other enteric pathogens like rotaviruses, coronaviruses, *Escherichia coli*, and *Cryptosporidium parvum*, which can complicate the clinical picture and increase disease severity [46,53].

Pathologically, natural BNeV infections cause intestinal lesions, mainly in the small intestine, characterized by villous atrophy, blunting, and fusion, with loss of absorptive epithelial cells. This damage impairs nutrient and fluid absorption, leading to diarrhea and malabsorption. In severe cases, the damage can be extensive enough to expose the underlying lamina propria. Experimental infections in Gn or colostrum-deprived calves with BNeV consistently reproduce these clinical signs and intestinal lesions, confirming their enteropathogenic nature [69]. Following oral inoculation, calves experimentally infected with BNeV consistently develop clinical signs similar to those seen in natural infections. Diarrhea, ranging from mild to severe and often watery in consistency, typically develops within 2 to 5 dpi. Other common clinical signs include anorexia, lethargy, and depression. Functional impairment of the intestine is often demonstrated by xylose malabsorption, indicating reduced absorptive capacity. Viral antigen is detectable in infected enterocytes. These studies have also documented the recovery of the intestinal mucosa in convalescent animals. Viral shedding in feces is common and can be prolonged. The severity of disease in natural infections varies due to factors such as coinfections, calf age, immune status, colostrum intake, and potential strain virulence differences. The primary pathological impact of BNeVs appears to be direct damage to intestinal enterocytes, leading to malabsorptive diarrhea. There is currently less evidence from the provided data to suggest significant systemic spread or substantial pathology outside the gastrointestinal tract for BNeVs, which contrasts with some other caliciviruses, such as certain vesiviruses known to cause systemic disease and multi-organ involvement.

### 3.3. Epidemiological Features

#### 3.3.1. Distribution, Prevalence

BNeV are globally distributed, detected in cattle populations across Europe, Asia, Africa, and Americas (Table 4) [42,44,46,47,48,50,67,70,71,72]. Virological prevalence studies, primarily using RT-PCR on fecal samples, show considerable variation in reported rates. Notably, these investigations typically target calves, especially those with clinical signs of diarrhea. This wide range is influenced by genuine epidemiological differences, as well as methodological variations between studies, such as diagnostic assay sensitivity and sampling strategies. For example, in Sweden, BNeV was found in 5% of sampled calves, affecting 16% of herds, whereas in France, 7% of diarrheic calves were positive [42,44]. Chinese studies reported prevalence rates up to 41.8% [50,67]. The prevalence rates in diarrheic calves were 5% in Tunisia, 4.8% in Brazil, 9.1% in South Korea, and 13.1% in Italy [48,70,71,72].

A common epidemiological feature is the frequent co-infection of BNeVs with other enteric pathogens, including BNoV, rotavirus, coronavirus, and bovine viral diarrhea virus (BVDV). For example, in the Swedish study, 6% of herds had evidence of both BNoV and BNeV infections [42], and in the French study, 2% of samples were positive for both viruses [44]. This co-occurrence complicates the assessment of each pathogen contribution to clinical disease severity in field settings, highlighting that neonatal calf diarrhea is often a multifactorial syndrome.

Serological studies, detecting antibodies, indicate past exposure to BNeV. While large-scale seroprevalence data for BNeV is less common, antibodies to NA1-like viruses are common in UK cattle, suggesting significant historical exposure [9]. BNeV infections primarily affect young, neonatal calves. A Swedish study found BNeV-infected calves had a median age of 21 days, older than BNoV-infected calves (7 days) [42]. This statistically significant age difference might suggest subtle variations in their transmission dynamics, windows of host susceptibility, or incubation periods, warranting further investigation. BNeV is generally not detected in adult cows or calves over 6 months [42]. Identifying specific risk factors has been challenging, though univariable analysis suggested associations with geographic location and early calf-dam separation.

#### 3.3.2. Zoonotic Potential

The investigation into the zoonotic potential of BNeV is less conclusive compared to other bovine enteric viruses. Molecular studies, specifically demonstrating that BNeV virus-like particles (VLPs) can bind to human histo-blood group antigens (HBGAs), suggest a potential for interaction with human cells [73]. However, humans primarily express type 1-chain based HBGAs in their gut mucosa, while cattle express type 2-chain based HBGAs [74]. This difference might represent a biological barrier limiting efficient human infection. Receptor binding is a prerequisite but not the sole determinant of successful zoonotic spillover.

Serological studies on human exposure to BNeV, particularly in occupationally exposed individuals, are needed, as detailed results from such surveys are not extensively covered. A study in France screened 110 human stool samples from gastroenteritis outbreaks, failing to detect any GIII BNoV or BNeV, despite their circulation in local calf populations [44].

## 4. Bovine Vesivirus Infections

In 1983, Smith and colleagues reported the isolation and characterization of a calicivirus from cattle that was subsequently classified within the *Vesivirus* genus [11]. This strain, BCV-Bos-1, also referred to as Tillamook virus, was isolated from calves in a dairy herd experiencing respiratory problems. This clinical presentation was distinct from the enteric diseases typically associated with other genera of bovine caliciviruses, i.e., *Norovirus* and *Nebovirus*. A crucial step in classifying BCV-Bos-1 as a member of the *Vesivirus* genus was the observation that inoculation into swine resulted in vesicular disease, a pathological hallmark of vesiviruses [11].

### 4.1. Molecular Biology and Classification

The genus *Vesivirus* forms a distinct group within the *Caliciviridae* [1]. A distinguishing feature of vesiviruses is that their second open reading frame (ORF2) encodes the major capsid protein (VP1) as a larger precursor, which is subsequently cleaved to produce the mature VP1; this precursor nature of VP1 appears to be unique to this genus. The vesivirus genome, typically 7.6 to 8.4 kb (Figure 1), is generally organized into three major ORFs: ORF1 encodes a polyprotein that is processed into non-structural proteins, ORF2 encodes the VP1 precursor, and ORF3 encodes the minor structural protein VP2 [1].

A key feature of the *Vesivirus* genus is its exceptionally broad host range, encompassing swine, cats, dogs, various marine mammals (such as sea lions, seals, and cetaceans), cattle, rabbits, primates (including humans), reptiles, and fish [75,76]. Marine vesiviruses, in particular, are known to be genetically closely related and generally lack strict host specificity [1]. The bovine vesiviruses strain BCV-Bos-1 (Tillamook virus) clusters in phylogenetic analyses with swine- and sea-lion-origin vesiviruses [77]. The molecular and pathobiological linkage underscores that bovine vesivirus infections are often not due to a uniquely bovine-adapted lineage but rather represent incursions of vesiviruses with a broad host and ecological range, frequently connected to the broader group of “marine vesiviruses.” Cattle can also be infected by other marine vesiviruses, likely through environmental exposure or the consumption of feed ingredients contaminated with material from marine origins [11,78]. Serological evidence further supports this observation, with antibodies to marine vesiviruses being detected in cattle populations [79]. Another vesivirus of relevance is Vesivirus 2117, which was identified as a contaminant in Chinese Hamster Ovary (CHO) cell bioreactors used in pharmaceutical manufacturing. Bovine serum has been implicated as a possible source of this contamination [80]. Phylogenetically, Vesivirus 2117 is closely related to canine vesiviruses [81].

### 4.2. Clinical Features and Pathology

Vesivirus infections in cattle have been linked to various clinical signs. A significant challenge in defining the precise clinical impact of vesiviruses in cattle stems from the overlapping symptomatology with other common bovine pathogens and the historical lack of specific diagnostic tests. Associations with abortion or respiratory disease are often based on serological data or limited virus isolations, rather than extensive controlled studies in cattle.

The Bos-1 strain (Tillamook virus) was originally isolated from calves with respiratory problems [11]. Some studies show statistical links between vesivirus infection and severe respiratory disease in cattle [79]. Marine vesiviruses, related to the bovine Bos-1 strain, cause vesicular lesions in susceptible hosts. Vesicular lesions are characterized by hydropic degeneration of epithelial cells, particularly in the stratum spinosum or stratum germinativum, leading to the formation of intraepidermal vesicles. These vesicles can rupture, leading to erosions and ulcers, accompanied by necrosis and an inflammatory cell infiltrate. While widespread outbreaks in cattle are not well-documented, the potential exists. Experimental inoculation of the Bos-1 strain into pigs produced characteristic vesicular disease. Clinical signs, often described for swine, include blisters in the oral cavity and on the feet, along with fever and lameness [11].

Evidence suggests an association between vesivirus infection and abortion in cattle [3]. A calicivirus (BCV Bos-2) similar to Vesicular Exanthema of Swine Virus (VESV) has also been detected in a spontaneously aborted bovine fetus. Serological investigations have also supported this association, with studies reporting higher prevalence of vesivirus antibodies or higher antibody titers in cattle that have aborted compared to non-aborting animals [79]. In addition, vesivirus infection in cattle has been associated with diarrhea [79]. Of note, sequence comparison of BCV Bos-1 and Bos-2 in small fragments of helicase and RdRp shows the highest sequence identity to a rabbit vesivirus isolate [82].

### 4.3. Epidemiological Features

Epidemiological studies of vesivirus in cattle primarily use serological surveys to detect past exposure, and, to a lesser extent, virological methods for active infection. Challenges in virological surveillance include transient shedding, low viral loads, and the genetic diversity of vesiviruses, which necessitates broadly reactive PCR assays [79].

#### 4.3.1. Serology

Serological surveys are the main method for assessing vesivirus exposure in cattle, using ELISAs with recombinant antigens. Historically, virus neutralization tests were also used, particularly for typing different strains. A US study found a 15.2% overall seroprevalence, with herd variations from 0% to 80% [79]. This study identified several risk factors associated with higher antibody prevalence, including older age of the animals, dairy versus beef cattle breeds, and certain reasons for sample submission to the diagnostic laboratory. Notably, in ELISA, stronger antibody responses were observed among older cattle and in cattle that had experienced abortion, compared to values from cattle submitted for respiratory tract disease or other reasons. Earlier work also reported neutralizing antibodies against SMSV-5 in Kansas cattle [83]. Low levels of antibodies to VESVs and SMSVs have been detected along the US West Coast, reinforcing the link to marine reservoirs.

It is important to interpret serological data with caution: seroprevalence indicates past exposure to the virus but does not necessarily confirm active infection or clinical disease at the time of sampling. However, high seroprevalence rates are generally indicative of endemic circulation of the virus within a population.

#### 4.3.2. Zoonotic Potential

**Figure 3:**
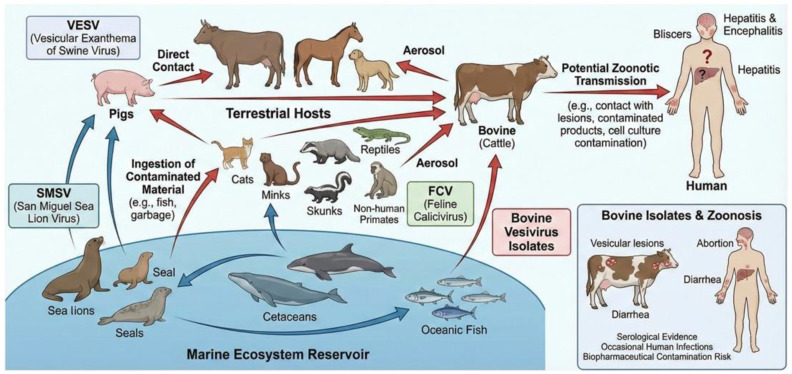
The complex ecological cycle of vesiviruses involving marine reservoirs, terrestrial hosts, and potential zoonotic transmission to humans. The typical clinical manifestations in bovine and human hosts are also highlighted.

The zoonotic potential of vesiviruses, including those found in cattle, is a significant concern due to their ability to cross species barriers and documented human infections. Vesiviruses, particularly from marine mammals, have a broad host range, infecting diverse species from fish to humans (Figure 3) [84].

Direct evidence of human infection includes a laboratory researcher who developed vesicular lesions after accidental SMSV-5 exposure, from which the virus was isolated [85]. Other cases involved a field biologist and the isolation of the SMSV-related Hom-1 strain from a human patient [74]. Vesivirus RNA has also been detected in human blood samples, with one study finding 9.8% positivity in human sera, including healthy donors and hepatitis patients [84]. Serological studies also indicate common human exposure, with antibodies to SMSV reported. A study found notable vesivirus antibody prevalence: 12% in healthy blood donors, 21% in those with elevated liver enzymes, 29% in hepatitis patients of unknown etiology, and 47% in hepatitis patients linked to transfusions or dialysis [84]. The vesivirus strain 2117 has been identified as contaminant of bioreactors used for production of human drugs, due to possible contamination of the reagents (likely bovine fetal serum) used for cell cultivation. Using ELISA, antibodies specific for 2117-like viruses have been detected in 32 of 410 (7.8%) human sera [86].

The presence of vesivirus antibodies in cattle and isolation of strains like Bos-1 and SMSV-5 from cattle, combined with their known zoonotic capabilities, support concerns about their zoonotic potential. Occupational exposure for agricultural workers, veterinarians, and slaughterhouse personnel could increase the risk of viral transmission.

## 5. Conclusions

Current knowledge indicates that bovine hosts harbor viruses representative of three *Caliciviridae* genera, i.e., noroviruses, neboviruses, and vesiviruses. There are significant genomic, genetic and marked antigenic differences among these three genera. Their clinical and epidemiological features also differ markedly. Noroviruses and neboviruses are primarily associated with watery diarrhea, whereas vesiviruses may induce diverse clinical signs including enteric, respiratory and vesicular disease.

Caliciviruses infecting bovine hosts are challenging to investigate due to their fastidious nature. The current knowledge of bovine infecting caliciviruses has been significantly shaped by the lack of a routine cell culture system, leading to a heavy reliance on molecular techniques for diagnosis and epidemiological surveillance, and on VLP-based assays for serology. When data are available, both virus detection and serology-based studies consistently demonstrate high rates of exposure in cattle populations worldwide, indicating that calicivirus infection is ubiquitous.

In this respect it is of note that while molecular techniques, particularly RT-PCR, have become the cornerstone of bovine calicivirus surveillance, the current diagnostic landscape is characterized by a lack of standardized performance metrics. Reported prevalence rates vary significantly between studies, partly due to differences in the sensitivity and specificity of various primers, some of which may fail to detect emerging recombinant or divergent strains. Furthermore, the applicability of these assays varies between clinical settings, where rapid pathogen identification is crucial for neonatal calf diarrhea management, and monitoring settings, which require broad-range detection for long-term epidemiological tracking. Future efforts should prioritize the comparative validation of diagnostic protocols to clarify their detection limits and clinical predictive value, ensuring that surveillance data are directly comparable across different geographical regions.

The zoonotic potential of caliciviruses harbored by cattle remains an area of active investigation. Regarding noroviruses, while cattle can be infected with and shed human-pathogenic norovirus strains (reverse zoonosis), there are no confirmed reports of bovine-specific GIII noroviruses causing clinical gastroenteritis outbreaks in humans despite BNoV-specific antibodies are present in some human populations. BNeVs have the molecular capacity to bind to certain human cell receptors in laboratory settings; however, screening of human stool samples from gastroenteritis outbreaks has failed to detect BNeV, even in areas where the virus is prevalent in cattle; thus, there is no evidence of human infection with BNeV. In this line, vesiviruses are confirmed zoonotic agents capable of causing clinical disease in humans; they can also contaminate biopharmaceutical products derived from bovine serum. Nonetheless, the specific role of cattle-derived vesiviruses in widespread human illness is not fully defined, as many human exposures are linked to marine reservoirs or unidentified sources.

Research into vaccines for BNoV and BNeV is in its early stages, with some efforts focusing on bivalent platforms to combat frequent co-infections in calves. One promising approach utilizes a baculovirus expression system to create bivalent VLPs that incorporate capsid proteins from both viruses. Preliminary results indicate that these VLPs successfully trigger both humoral and cellular immune responses in mouse models [87]. Another approach, a monovalent BNoV VLP vaccine candidate induced high serum IgG and IgA titers and activated cellular immune response in cattle [88]. Additionally, a bivalent human adenovirus vector vaccine has been developed to encode the VP1 genes of both bovine pathogens for delivery via oral or intramuscular routes. Testing of this adenovirus-based candidate showed significant antibody production and T-cell activation in mice, as well as robust immune responses in calves [89].

The significant knowledge gaps and challenges require concerted efforts for solutions. Thus, future research should prioritize several key areas, including (i) development of in vitro culture systems that permits studies on viral replication, pathogenesis, antiviral drug screening, and the development of conventional vaccines, (ii) improved understanding of transmission dynamics and subclinical infection, (iii) enhanced surveillance for emerging strains with a special focus on the emergence of novel recombinant strains, and (iv) assessing any changes in zoonotic risk and (v) overall, the clarification of etiological role of different bovine caliciviruses to multifactorial clinical diseases under field conditions.

## Figures and Tables

**Table 1:** Prototypes and representative bovine strains of noroviruses, neboviruses, and vesiviruses.

Designation	Year	Country	Current ICTV Classification (Family, Genus, Species)	References
Jena virus (Bo/Jena/DE/1980/GIII.1)	1980	Germany	*Caliciviridae*, *Norovirus*, *Norovirus norwalkense*	[4,5,6]
Newbury agent-2 (Bo/Newbury2/UK/1976/GIII.2)	1976	United Kingdom	*Caliciviridae*, *Norovirus*, *Norovirus norwalkense*	[7]
Newbury agent-1 (Bo/Newbury1/UK/1976)	1976	United Kingdom	*Caliciviridae*, *Nebovirus*, *Nebovirus newburyense*	[8,9]
Nebraska virus (Bo/Nebraska/US/1980)	1980	USA	*Caliciviridae*, *Nebovirus*, *Nebovirus newburyense*	[10]
Tillamook virus (BCV-Bos1)	1983	USA	*Caliciviridae*, *Vesivirus*, *Vesivirus exanthema*	[11]

**Table 2:** Comparison of clinical and pathological features between GIII.1 and GIII.2 BNoVs.

Feature	BNoV GIII.1	BNoV GIII.2
Host	Newborn Gn/conventional calves	Newborn Gn/conventional calves
Severity of diarrhea	Severe, watery	Mild to none in conventional calves Mild, intermittent but persistent in Gn calves
Onset of diarrhea	14–16 h post-inoculation	Variable; can be 1 day post-inoculation in Gn calves
Duration of diarrhea	~2–3 days (53–67 h)	Can persist for up to 20–26 days in Gn calves
Intestinal lesions	Severe villus atrophy, epithelial loss or attenuation, villous fusion (primarily jejunum/ileum); crypt hyperplasia	Minimal to mild lesions in some Gn studies; less severe or absent villous atrophy reported with some strains
Viral shedding	Shorter duration (e.g., fecal shedding stops up to 4 dpi)	Prolonged fecal RNA shedding (e.g., fecal shedding may last 20–30 dpi)

**Table 3:** Epidemiological features of BNoV infections.

Continent	Country	Period	Age	Health Status	Methodology	Detection Rate	Cite
Europe	Belgium	2007	<6 m	Diarrheic	RT-PCR	7.5% (10/133)	[38]
	France	2005–2008	Mean age, 9 d	Diarrheic	RT-PCR	20% (89/456)	[44]
		2010	<1 m	Diarrhea	RT-PCR	30.9% (25/81)	[43]
	Hungary	2002, 2008	All ages (2002); <1 m (2008)	Healthy	RT-PCR	8.5% (4/47) in 2002; 3.8% (1/26) in 2008	[39]
	Italy	2004–2005	<1 m	Diarrheic	RT-PCR	20.8% (21/101)	[40]
		2011–2012	<2 m	Asymptomatic	RT-nPCR	10.5% (11/104)	[41]
	Sweden	2005–2012	<3 m	Calves with and without diarrhea	RT-PCR	19% (cross-sectional); 28% (longitudinal)	[42]
	UK	1998–2000	All ages	Diarrheic	RT-PCR	11% (44/398)	[37]
Asia	China	2016–2017	3–4 m	Diarrheic	RT-PCR	10.7% (3/28)	[50]
		2017-2018	<3 m	Diarrheic	RT-PCR	20.4% (43/211)	[22]
		2021–2023	Not specified	Diarrheic	qPCR	0.68% (2/295)	[51]
		2022–2024	Adult	Not specified	nPCR; NGS	15.98% (31/194)	[52]
	Iran	2010–2012	<1 m	Diarrheic	RT-PCR	39.5% (100/253)	[47]
	South Korea	2004–2005	<3 m	Diarrheic	RT-PCR, nPCR	9.3% (60/645)	[45]
	Türkiye	2002–2016	<7 m	Diarrheic	RT-nPCR	33.5% (56/167)	[46]
Africa	Egypt	2015	<10 m	Diarrheic	RT-PCR	24% (6/25)	[49]
	Tunisia	2006–2010	<3 m	Diarrheic	RT-PCR	16.6% (28/169)	[48]
Americas	Argentina	2008–2012	<3 m	Diarrheic	RT-PCR; NGS	3.3% (3/90)	[36]
	USA		<1 m	Diarrheic	RT-PCR	80% (48/60) in Michigan; 33% (4/12) in Wisconsin	[34]
	Uruguay	2015–2018	<2 m	Diarrheic and non-diarrheic 76.2%	RT-qPCR	66.1% (503/761)	[35]

Note: Reported prevalence ranges are not directly comparable due to study design differences.

**Table 4:** Epidemiological features of BNeV infections.

Continent	Country	Period	Age	Health Status	Methodology	Detection Rate	Reference
Europe	France	2005–2008	Mean 9 d	Diarrheic	RT-PCR	7% (34/456)	[44]
	Italy	2009–2010	<2 m	Diarrheic and non-diarrheic	RT-nPCR	0% non-diarrheic; 13.1% diarrheic	[71]
	Sweden	2005–2012	<3 m	Diarrheic and non-diarrheic	RT-PCR	4.5% (cross-sectional); 10% (outbreaks)	[42]
Asia	China	2016–2017	3–4 m	Diarrheic	RT-PCR	10.7% (3/28)	[50,67]
		2017–2018	<4 m	Diarrheic and non-diarrheic	RT-PCR	41.8% (diarrheic); 5.7% (non-diarrheic)	
	Iran	2010–2012	<1 m	Diarrheic	RT-PCR	15% (38/253)	[47]
	South Korea	2004–2005	<3 m	Diarrheic	RT-PCR; nPCR	9.1% (59/645)	[72]
	Türkiye	2002–2016	<7 m	Diarrheic	RT-nPCR	22.1% (37/167)	[46]
Africa	Tunisia	2006–2010	<3 m	Diarrheic	RT-PCR	3.0% (5/169)	[48]
Americas	Brazil	2012–2013	<4 m	Diarrheic and non-diarrheic	RT-PCR	4.8% (3/62)	[70]

Note: Reported prevalence ranges are not directly comparable due to study design differences.

## Data Availability

No new data were created or analyzed in this study. Data sharing is not applicable to this article.
